# Activating Attachments Reduces Memories of Traumatic Images

**DOI:** 10.1371/journal.pone.0162550

**Published:** 2016-09-15

**Authors:** Richard A. Bryant, Rachael Foord

**Affiliations:** University of New South Wales, Sydney, Australia; Technion Israel Institute of Technology, ISRAEL

## Abstract

Emotional memories, and especially intrusive memories, are a common feature of many psychological disorders, and are overconsolidated by stress. Attachment theory posits that activation of mental representations of attachment figures can reduce stress and boost coping. This study tested the proposition that attachment activation would reduce consolidation of emotional and intrusive memories. Sixty-seven undergraduate students viewed subliminal presentations of traumatic and neutral images, which were preceded by subliminal presentations of either attachment-related images or non-attachment-related images; free recall and intrusive memories were assessed two days later. Participants with low avoidant attachment tendencies who received the attachment primes recalled fewer memories and reported fewer intrusions than those who received the non-attachment primes. Unexpectedly, those with high anxious attachment tendencies reported fewer memories. These findings generally accord with attachment theory, and suggest that consolidation of emotional memories can be moderated by activation of attachment representations.

## Introduction

Emotional memories, and particularly intrusive memories are a common feature of many psychological disorders [1]. Most models of emotional memories converge on the critical role of arousal at the time of memory consolidation that contributes to subsequent distressing memory [2, 3]. Biological models propose that memory traces are strengthened by activation of glucocorticoid receptors in the basolateral nucleus of the amygdala facilitating noradrenergic signals [4]. Supporting this proposition is much evidence that strength of emotional memories is moderated by glucocorticoid and noradrenergic activation at the time of consolidation [5, 6]. Further support comes from literature on intrusive emotional memories, which occur involuntarily and are typically vivid and distressing [7], and that are also associated with elevated levels of both glucocorticoid and noradrenergic response [8].

There is distinct research suggesting that proximity to attachment figures alleviates stress responses [9, 10]. This evidence reflects a fundamental tenet of attachment theory that during stress we seek social support [11]. This theory posits that as one develops, one internalizes mental representations of attachment figures, and these similarly can provide a sense of security during stress. Supporting this proposition is much evidence that people with secure attachments have a better capacity to manage stress [12, 13]. Further, experimentally activating attachment figures results in a range of psychological benefits, including reduced bias to threat [14] and reduced pain-related neural activation [15].

The extent to which attachments can benefit people appears to depend, however, on individual differences in attachment security. Attachment theories posit that prior experiences of unreliable relationships can lead to deficiencies in the capacity to benefit from activation of attachment representations because this does not promote a sense of security [16]. Insecure attachments can result in people having anxious or avoidant attachment tendencies. People with anxious attachments tend to *hyperactivate* their attachment system during stress because there is an urgent need for calming from others. In contrast, those with avoidant attachment tendencies will *hypoactivate* their attachment system and distance themselves from others during threat as a means of coping. Supporting this proposal is evidence that during threat, avoidantly attached individuals inhibit proximity-seeking behavior [17] and are less likely to activate attachment representations [18].

Despite the evidence that attachments can ameliorate stress responses, no research has directly addressed the extent to which attachments may limit emotional memories. There is indirect evidence of a link between attachment and emotional memories from literature on posttraumatic stress disorder (PTSD), which is characterized by intrusive memories of a traumatic event. Secure attachments have been shown to mitigate PTSD symptoms after trauma [19]. Further, providing attachment primes to participants with PTSD results in reductions in attentional bias to threats, suggesting that attachment representations can ameliorate one of the core dysfunctions in PTSD [14]. To test the potential impact of attachments on intrusive memories directly, the current study presented participants with subliminal primes of attachment or non-attachment figures immediately prior to presentation of trauma-related and neutral stimuli. Participants were then assessed two days later to index occurrence of intrusive memories of the presented stimuli.

## Method

### Participants

Sixty-seven (23 female) undergraduate psychology students of mean age 19.78 years (*SD* = 2.31) at the University of New South Wales were recruited in return for course credit. Sixty-three participants completed the follow-up memory tests (31 in Attachment and 32 in non-Attachment conditions). Following previous study demonstrating moderate effect of attachment activation studies using subliminal primes [17], we estimated a required sample size of at least 30 per cell to achieve an effect size of 0.7 between the two conditions, providing power of 80% to detect a difference between conditions at the 5% significance level. We recruited participants till we obtained 31 in each cell (to allow for missing data in some participants).

### Measures

#### Experiences in Close Relationships scale (ECR; [20])

The ECR is a 36-item self-report questionnaire that measures adult attachment along the two dimensions of attachment anxiety and avoidance. The anxious and avoidant subscales of this measure have a high level of internal consistency, with coefficient alphas of .91 and .94, respectively [21]. The ECR asks participants to rate on a 7-point Likert scale the extent to which they agree with the statements pertaining to how they feel in emotionally intimate relationships. The statements in the original version of the ECR ask participants how they feel in their romantic relationships. Following prior use of this scale with more generic use [22], the words “romantic partner” in the original statements were altered to “the person who I consider close to me”.

#### Depression Anxiety Stress Scale

The DASS-21 is a 21-item self-report questionnaire that measures the severity of depression, anxiety and stress levels over the past week. Items are scored on a 0 to 3-point scale (0 = *did not apply to me at all*, 3 = *applied to me very much or most of the time*). The DASS-21 total score and subscales have high internal consistencies ranging from .82 to .93 in a non-clinical sample [23]. Cronbach’s alpha for the total score and subscales in the current sample ranged from .84 to .93.

#### Impact of Event Scale-Revised

The IES-R is a 15-item self-report questionnaire that indexes intrusive, avoidance, and arousal responses to a traumatic event. [IES; 24]. Items are scored on a 0 to 4-point scale (0 = *not at all*, 4 = *extremely*). For the purpose of indexing intrusive memories of the stimuli presented in this experiment, we used selected items from the Intrusion subscale that directly pertained to the occurrence of unwanted memories of the target event (e.g ‘Pictures about it popped into mind’).

### Procedure

This study was approved by the University of New South Wales School of Psychology Research Ethics Committee. Participants initially completed written informed consent, and then completed the ECR and DASS-21. Anxiety and depression were assessed with the e Depression Anxiety Stress Scale (DASS-21; [25]).

Participants were then directed to sit in front of a computer and were informed that they were to attend to the screen, which would present a variety of images. Using DirectRT^™^ (Empirisoft, version 2014), 40 images from the International Affective Picture System [26] were presented to the participants for 5 sec each. Twenty of the images were trauma-related in content (e.g. scenes of war, violence, bloody or dismembered bodies; mean arousal rating: 6.13, mean valence rating: 4.96), and 20 of the images were neutral in valence (e.g. landscapes, household items, furniture; mean arousal rating: 2.66, mean valence rating: 2.85). The images were presented in 8 blocks of 5 images according to valence. All images were randomly presented within blocks, and all blocks were randomized (whilst alternating between trauma-related and neutral images).

Prior to each image, participants were subliminally primed with one of five images (randomly selected); participants in the Attachment condition were primed with attachment-related images (e.g. photographs of a mother or father holding a baby) and those in the Non-Attachment condition were primed with non-attachment-related images (e.g. individuals with pleasant facial expressions) with the gender and ethnicity differences matched across the two sets. Primes appeared for 17ms before each image, followed by a backwards mask for 500ms.

Thirteen additional participants were recruited to validate the subliminal paradigm. These participants were informed that an image would appear on a computer display unit, followed by a geometric pattern (these images involved the 17ms stimuli followed by the backwards mask for 500ms). Two images were presented, one on each side of the screen, and participants indicated using the keyboard which image they thought they had been initially shown. Participants viewed all ten primes in the actual experiment twice in a randomized order. Participants correctly identified both attachment-related primes (*M* = 5.769/10) and non-attachment related (*M* = 5.231/10) primes at chance rates.

After each image, participants were asked to indicate on the keyboard how uncomfortable they felt whilst viewing the picture on a 7-point Likert scale (1 = ‘not at all uncomfortable’, 7 = ‘extremely uncomfortable’). After entering their rating, a fixation cross appeared on the screen for 1 second, and then the next prime-mask-image series was presented. Three practice items occurred at the beginning of the experiment in order to demonstrate the procedure.

After all 40 images were presented participants the experimental session was terminated. Participants were informed that they would be followed up in two days via an email, with a link to an online questionnaire that aimed to collect their feedback on the study (they were informed to complete the questionnaire within 24 hours of receiving the link, and that extra credit would be granted to them). Participants were not informed at this stage that they would be administered any memory tests for the experimental session.

Two days later, participants were emailed a link to an online questionnaire. Participants completed the Intrusions subscale of the Impact of Event–Revised scale in relation to the stimuli that were presented during the experimental session [27].

### Data Analysis

To determine the potential impact of being highly avoidant or anxious on the attachment scale (ECR) on the dependent variables, we conducted separate analyses of variance (ANOVA) that factored in high and low scores on the anxious attachment and avoidant attachment dimension, respectively. Prior to analyses, Kolmogorov-Smirnov analyses were conducted to determine normal distributions. Median splits were calculated to determine high and low scorers on the ECR anxious attachment and avoidant attachment scales, respectively. The median spit was 67/68 (low anxious range: 24–67; high anxious range: 68–107) for ECR-Anxious was 45/46 (low avoidant range: 24–45; high avoidant range: 46–92) was ECR-Avoidant. Although attachment style can be analyzed dimensionally, we used a medium split analysis because the modest sample size limited the sensitivity of continuous assessment of the attachment style in this study.

## Results

### Participant Characteristics

Mean participant characteristics are presented in Table 1. Participants in the two conditions did not differ in terms of age (Attachment: *M* = 18.53, *SD* = 1.02; Non-Attachment: *M* = 19.09, *SD* = 2.43), ECR Anxious Attachment (Attachment: *M* = 65.41, *SD* = 18.16; Non-Attachment: *M* = 67.00, *SD* = 17.49), ECR Avoidant Attachment (Attachment: *M* = 46.81, *SD* = 15.62; Non-Attachment: *M* = 46.44, *SD* = 14.48), DASS Depression (Attachment: *M* = 3.00, *SD* = 2.43; Non-Attachment: *M* = 2.63, *SD* = 2.43) or DASS Anxiety (Attachment: *M* = 2.65, *SD* = 2.58; Non-Attachment: *M* = 3.00, *SD* = 2.73) scores.

**Table 1:** Mean Ratings of Images.

	Avoidant Attachment			
	High		Low	
	Attachment	Non-Attachment	Attachment	Non-Attachment
Negative	5.01 (1.05)	5.19 (1.06)	4.51 (1.48)	4.50 (1.26)
Neutral	1.47 (0.56)	1.84 (1.45)	1.27 (0.32)	1.48 (0.50)
	Anxious Attachment			
	Attachment	Non-Attachment	Attachment	Non-Attachment
Negative	4.67 (1.47)	4.76 (1.16)	4.78 (1.23	5.01 (1.27)
Neutral	1.28 (0.28)	1.88 (1.44)	1.42 (0.54)	1.39 (0.25)

*Note*. Standard deviations appear in parentheses.

### Image Ratings

Mean image ratings scores are reported in Table 1. In terms of high and low scorers on avoidant attachment, a 2 (Attachment Condition) x 2 (Avoidant Attachment) x 2 (Image Valence) ANOVA of image ratings indicated a significant main effect for Valence [*F* (1, 60) = 429.22, *p* = .000]. For anxious attachment, there was a significant main effect for Valence [*F* (1, 60) = 414.44, *p* = .000]. Ratings of negative images were rated as more negative than neutral images. There were no interaction effects.

### Free Recall

Mean recall scores are reported in Table 2. In terms of high and low scorers on avoidant attachment, a 2 (Attachment Condition) x 2 (Anxious Attachment) x 2 (Memory Valence) ANOVA of recall responses indicated a significant main effect for Valence [*F* (1, 56) = 48.22, *p* = .04], and a significant Attachment Condition x Anxious Attachment interaction [*F* (1, 56) = 4.27, *p* = .000]. More negative words were recalled than neutral words. Posthoc analyses indicated that whereas participants with low avoidant attachment recalled fewer memories if they had the attachment primes relative to the non-attachment primes (*p* < .05), this difference was not observed in those with high avoidant attachment. In terms of high scorers on anxious attachment, there was a significant main effect for Valence [*F* (1, 56) = 43.67, *p* = .000], and a significant Attachment Condition x Anxious Attachment interaction, *F* (1, 56) = 5.07, *p* = .03. Whereas participants with low anxious attachment displayed comparable recall across attachment and non-attachment prime conditions, those with high anxious attachment recalled fewer memories if they had the attachment primes relative to the non-attachment primes (*p* = .01).

**Table 2:** Mean Recall Responses.

	Anxious Attachment			
	High		Low	
	Attachment	Non-Attachment	Attachment	Non-Attachment
Negative	2.58 (1.97)	4.06 (1.88)	4.51 (1.48)	4.50 (1.26)
Neutral	1.47 (0.56)	1.84 (1.45)	1.27 (0.32)	1.48 (0.50)
Intrusions	.67 (1.61)	1.87 (1.75)	.89 (1.50)	1.00 (2.42)
	Avoidant Attachment			
	Attachment	Non-Attachment	Attachment	Non-Attachment
Negative	4.57 (2.14)	3.59 (2.27)	2.37 (1.82)	3.46 (2.44)
Neutral	1.79 (1.42)	1.64 (1.32)	.87 (1.36)	1.77 (1.42)
Intrusions	1.29 (1.82)	1.00 (1.37)	.37 (1.09)	2.08 (2.72)

*Note*. Standard deviations appear in parentheses.

### Intrusive Memories

A 2 (Attachment Condition) x 2 (Anxious Attachment) ANOVA indicated no significant main or interaction effects (see Table 2). A 2 (Attachment Condition) x 2 (Avoidant Attachment) ANOVA indicated a significant Attachment Condition x Avoidant Attachment interaction effect, *F* (1, 56) = 4.57, *p* = .03. Specifically, participants with low avoidant attachment reported fewer subsequent intrusive memories when an attachment prime was presented relative to a non-attachment prime (*p* < .05). That is, fewer intrusive memories occurred after being primed with an attachment prime but only for participants with low avoidant attachment tendencies.

## Discussion

Priming words with attachment representations led to fewer memories being recalled and also reported fewer intrusive distressing memories if participants displayed low levels of avoidant attachment tendencies. Contrary to our hypothesis, we did not find a differential impact of attachment on negative and neutral words (even though more negative words were recalled than neutral words). It is possible that the immunizing effect of the attachment primes generalized from each word presentation to the next, and so the effect was found for both negative and neutral words.

Attachment theory proposes that those with a secure attachment style can benefit from activating attachment representations because they trigger the sense that one has the security of supported others, whom can protect them at times of need [28]. We hypothesized that the protective effect of attachments would impact on stress responses by down-regulating the arousal that occurs at the time of memory consolidation [3]. Numerous studies have previously found that memory consolidation can be impaired by pharmacological means to interrupt the glucocorticoid-noradrenergic surge that underpins memory consolidation [29]. This study shows that attachment activations can play a comparable role by possibly reducing consolidation. We did not index stress hormones or heart rate variability, so we cannot conclude the extent to which attachment activation directly impacted on these processes. Moreover, we did not find that participants’ ratings of the images were moderated by whether they received the attachment or non-attachment prime, which does not support the interpretation that attachment priming ameliorated the affective response to images. It also possible, however, that the effects of the subliminal attachment prime may not have been reflected in participants’ conscious response to the images.

A curious finding was that participants with high anxious attachment reported fewer memories if they received the attachment primes than if they received the non-attachment prime. On face value this finding seems inconsistent with the proposal of attachment theory that although people who are anxiously attached will seek proximity to attachments during stress, this attempt will be not provide security because they fear that attachments are not readily accessible [30]. Although some prior work has shown that anxious attachment is associated with increased cortisol response to stressors [31], other work has shown that anxious attachment is associated with decreased cortisol response when these people are also avoidantly attached [32]. It is apparent that the relationship between cortisol response and response to stress is not linearly related to the avoidant/anxious dimensions. This conclusion is underscored by evidence that there are different interactions of attachment availability and attachment style in terms of psychological and neuroendocrine responses[10]. It is also possible, however, that because participants in this study were healthy undergraduate students (rather than a clinical population), anxiously attached individuals may have responded more to the attachment than non-attachment cues by hyperactivating their attachment system, and this provided them with greater benefit and reduced arousal relative to those who received the non-attachment primes. This in turn may have led to weaker memory consolidation in anxiously attached individuals who received the attachment primes.

Noteworthy in this study is the fact that the effects of attachment primes on subsequent memory occurred at a preconscious level. Prior studies have shown that subliminal presentations of attachment primes can minimize information processing of threat [17], reduce self-blame [33], enhance intimate behavior [34], positive ratings of neutral stimuli [35], and reduce threat bias in PTSD [14]. The current finding indicates that activating attachment representations at a preconscious level can also impact how memories are consolidated. This suggests that the stage at which this influence is exerted is very early in the processing of the attachment, and highlights that people can be highly attuned to these cues.

We note several limitations to the study. First, the lack of hormonal and psychophysiological data limits our inferences about how attachment activation may impact on the stress response system. Second, our measure of intrusive memories relied on retrospective reporting, and it is possible that diary [36] or momentary analysis recording [37] would have provided a more accurate index of intrusions. Third, activating attachments via presentation of standardized images may lack self-referential relevance for participants; prior studies that have used names or images of people who are actual attachment figures of participants have reported strong effects [35], and could be considered in future replication. Fourth, other measures of attachment are available, including the Adult Attachment Interview [38] and the Adult Attachment Projective [39], which may provide more nuanced indices of attachment style. Finally, we note that our sample size limited the extent to which we could assess the impact of the attachment prime on certain subtypes of attachment style. Numerous researchers have noted a range of attachment styles beyond the dichotomony of avoidant/attachment dimensions [40]; for example, a larger sample would have allowed more nuanced examination of participants with different permutations of attachment style (e.g. high anxious and high avoidant tendencies).

This study provides the first evidence that activating attachment representations can significantly affect how people consolidate trauma-related memories. Moreover, the extent to which this effect occurs is moderated by people’s attachment style. Considering that social support has been shown to reduce the development of fear-based memory disorders after stressors [41, 42], this finding may shed light on one mechanism by which emotional memories are ameliorated in the aftermath of trauma. These findings may also offer direction for future research into how psychotherapies, including attachment-related psychotherapy, can impact on posttraumatic responses.

## Supporting Information

S1 Dataset Participant characteristics and behavioral data. (SAV)
